# Comparison of oral sodium phosphate tablets and polyethylene glycol lavage solution for colonoscopy preparation: a systematic review and meta-analysis of randomized clinical trials

**DOI:** 10.3389/fmed.2023.1088630

**Published:** 2023-05-26

**Authors:** Li Yao-dong, Wang Yi-ping, Mai Gang, Han Yang-yun, Zhu Ling-ling, Deng Hong, Deng Jia-zheng, Xiang Rong-chao, Li You-wei, Zhao Ming, Ding Shun-bin, Ling Jing, Shen Yang, Dong Jia-qi, Deng Lei, Song Xiong-feng, Zhang You-jian, Zhou Zuo-qiong

**Affiliations:** ^1^Gastroenterology Department of Deyang People's Hospital, Deyang, China; ^2^School of Medical and Life Sciences, Chengdu University of Traditional Chinese Medicine, Chengdu, China; ^3^West China Hospital, Sichuan University, Chengdu, China; ^4^Surgical Department of Deyang People's Hospital, Deyang, China

**Keywords:** colonoscopy, bowel preparation, sodium phosphate, polyethylene glycol electrolyte lavage solution, meta-analysis, systematic review

## Abstract

**Objective:**

To systematically compare the bowel cleaning ability, patient tolerance and safety of oral sodium phosphate tablets (NaPTab) and oral polyethylene glycol electrolyte lavage solution (PEGL) to inform clinical decision making.

**Methods:**

PubMed, Embase, CBM, WanFang Data, CNKI, and VIP databases were searched for studies that used randomized controlled trials (RCTs) to compare the roles of NaPTab and PEGL in bowel preparation before colonoscopy. Two reviewers independently screened the studies, extracted data, and assessed the risk of bias in the included papers. A meta-analysis was performed using RevMan 5.3 software.

**Results:**

A total of 13 RCTs were eligible for inclusion, including 2,773 patients (1,378 and 1,395 cases in the NaPTab and PEGL groups, respectively). Meta-analysis revealed no significant difference in the cleansing quality of the NaPTab and PEGL groups [RR 1.02, 95% CI (0.96–1.08), *P* = 0.46]. The incidence of nausea was lower in the NaPTab group than in the PEGL group [RR 0.67, 95% CI (0.58–0.76), *p* < 0.00001]. Patients rated the taste of NaPTab higher than PEGL [RR 1.33, 95% CI (1.26–1.40), *P* < 0.00001]. Willingness to repeat the treatment was also higher in the NaPTab group than in the PEGL group [RR 1.52, 95% CI (1.28–1.80), *P* < 0.00001]. Both serum potassium and serum calcium decreased in both groups after the preparation; however, meta-analysis revealed that both minerals decreased more in the NaPTab group than in the PEGL group [MD = 0.38, 95% CI (0.13–0.62), *P* = 0.006 for serum potassium and MD = 0.41, 95% CI (0.04–0.77), *P* = 0.03 for serum calcium]. Meanwhile, serum phosphorus increased in both groups after the preparation; however, levels increased more in the NaPTab group than in the PEGL group [MD 4.51, (95% CI 2.9–6.11), *P* < 0.00001].

**Conclusions:**

While NaP tablets and PEGL were shown to have a similar cleaning effect before colonoscopy, NaP tablets had improved patient tolerance. However, NaP tablets had a strong effect on serum potassium, calcium, and phosphorus levels. For patients with low potassium, low calcium, and renal insufficiency, NaP tablets should be prescribed with caution. For those at high-risk for acute phosphate nephropathy, NaP tablets should be avoided. Given the low number and quality of included studies, these conclusions will require additional verification by large high-quality studies.

**Systematic review registration:**

10.37766/inplasy2023.5.0013, identifier: NPLASY202350013.

## 1. Introduction

Ideal bowel preparation allows for a detailed examination of the entire colon and should be safe and acceptable to patients. Insufficient bowel preparation is a cause of incomplete colonoscopy. Polyethylene glycol electrolyte lavage solution (PEGL), for example, is associated with inadequate cleaning rates of ≥10% (1, 2). Insufficient bowel preparation before colonoscopy can prevent the cecum from being reached or even approached and cause the mucous membranes to appear unclear (3). There is no ideal preparation that is safe, convenient, tolerable, and inexpensive (4). Oral sodium phosphate (OSP) and PEGL are the main methods for bowel preparation (5). PEGL has been available since 1980 and its efficacy was established compared with older diet and cathartic regimens (6). PEGL are large-volume (2–4 L), osmotically-balanced non-absorbable solutions that act as purgatives to evacuate the intestine. Similarly, 2 L PEG/bisacodyl preparations are as effective as the standard 4 L PEG regimens but are better tolerated (7). OSP acts as an osmotic purgative, drawing water into the bowel lumen and stimulating peristalsis and evacuation and OSP solution is a low volume laxative (8).

Oral sodium phosphate tablets (NaPTab) are convenient and associated with a lower incidence and severity of nausea than OSP solution (9). The current study conducted a meta-analysis of reports conducted to compare the use of NaPTab and PEGL for colonoscopy preparation. Differences in bowel cleaning effect, patient tolerance and safety were assessed to inform strategies for clinical use of the two treatments.

## 2. Materials and methods

### 2.1. Registration and search strategy

#### 2.1.1. Registration

The registration number of the systematic review protocol was INPLASY202350013 in the INPLASY.

#### 2.1.2. Search strategy

Preferred Reporting Items for Systematic Reviews and Meta-Analyses (PRISMA) guidelines were used to conduct this meta-analysis (10). A systematic search was performed of several databases, including PubMed, China National Knowledge Infrastructure (CNKI), Chinese Science and Technology Journals Database (VIP), Chinese Biomedical Literature Database (CBM), Wanfang Database, and Embase. The search was conducted using the following keywords: colonoscopy, bowel preparation, bowel cleaning, sodium phosphate, sodium phosphate tablets, polyethylene glycol electrolyte lavage solution, and polyethylene glycol. The retrieval strategy is shown in Box 1 using PubMed as an example.

Box 1 **PubMed retrieval strategy.**

#1 colonoscopy

#2 bowel preparation OR bowel cleaning

#3 sodium phosphate OR sodium phosphate tablets

#4 polyethylene glycol electrolyte lavage solution OR polyethylene glycol

#5 #1 AND #2 AND #3 AND #4

### 2.2. Inclusion criteria

The inclusion criteria included (1) patient populations with an indication for colonoscopy, including outpatients or inpatients requiring diagnosis or treatment (those receiving a sigmoidoscopy were not considered), (2) randomized controlled trial (RCT) study designs, (3) a sodium phosphate tablet intervention group, (4) a control group receiving PEGL administered orally or by nasogastric tube, (5) outcome measures including cleansing quality, adverse effects (incidence of nausea), patient acceptance (taste and willingness to repeat the treatment), and changes in serum electrolytes after preparation, and (6) all included articles were available in English.

### 2.3. Exclusion criteria

The exclusion criteria included (1) articles that were not rigorous or had incomplete data, (2) trials of bowel preparation treatments other than NaPTab and PEGL, (3) repeated publication of the same study, and 4)not in the English language.

### 2.4. Data extraction and quality assessment

Two reviewers (Li Yao-dong and Zhou Zuo-qiong) independently screened the literature, extracted data and cross-checked studies that met both the inclusion and exclusion criteria. Differences were solved by discussion or consultation with a third reviewer (Zhu Ling-ling et al.). The researchers began by reading the titles of the articles and excluding any studies that were obviously irrelevant before reading the abstract or full text of remaining articles to determine whether or not they should be included. Extracted data primarily included: basic report information (including title, author, publication date), methodology (including sample size, baseline characteristics, interventions), outcome evaluation indicators (including adverse drug events and adverse drug reactions) and key elements associated with a risk of bias.

Quality assessment was performed using the Cochrane risk-of-bias tool, which consists of a random distribution method, a distribution hiding method, a blind method, incomplete results and other deviations. Each study was rated as “yes” (low risk of bias), “no” (high risk of bias) or “unclear” (uncertain risk of bias) (11).

### 2.5. Statistical analysis

Meta-analysis was conducted using RevMan5.3 software. Quantitative data were presented as the relative risk degree (RR) and 95% confidence interval (CI). Measurement data included the mean difference (MD) and 95% CI as the effect index. The chi-square and I-square tests were used to measure heterogeneity in the trials (the test level was α = 0.05). If there was no obvious heterogeneity (*p* > 0.10, *I*^2^ < 50%) then the fixed effect model was used for analysis. When *p* < 0.10 and *I*^2^ ≥ 50%, a random effect model was used for analysis. Sensitivity analysis were used to handle obvious clinical heterogeneity. Publication bias was assessed through the visual check of funnel plots.

## 3. Results

### 3.1. Study selection process

**Figure 1:**
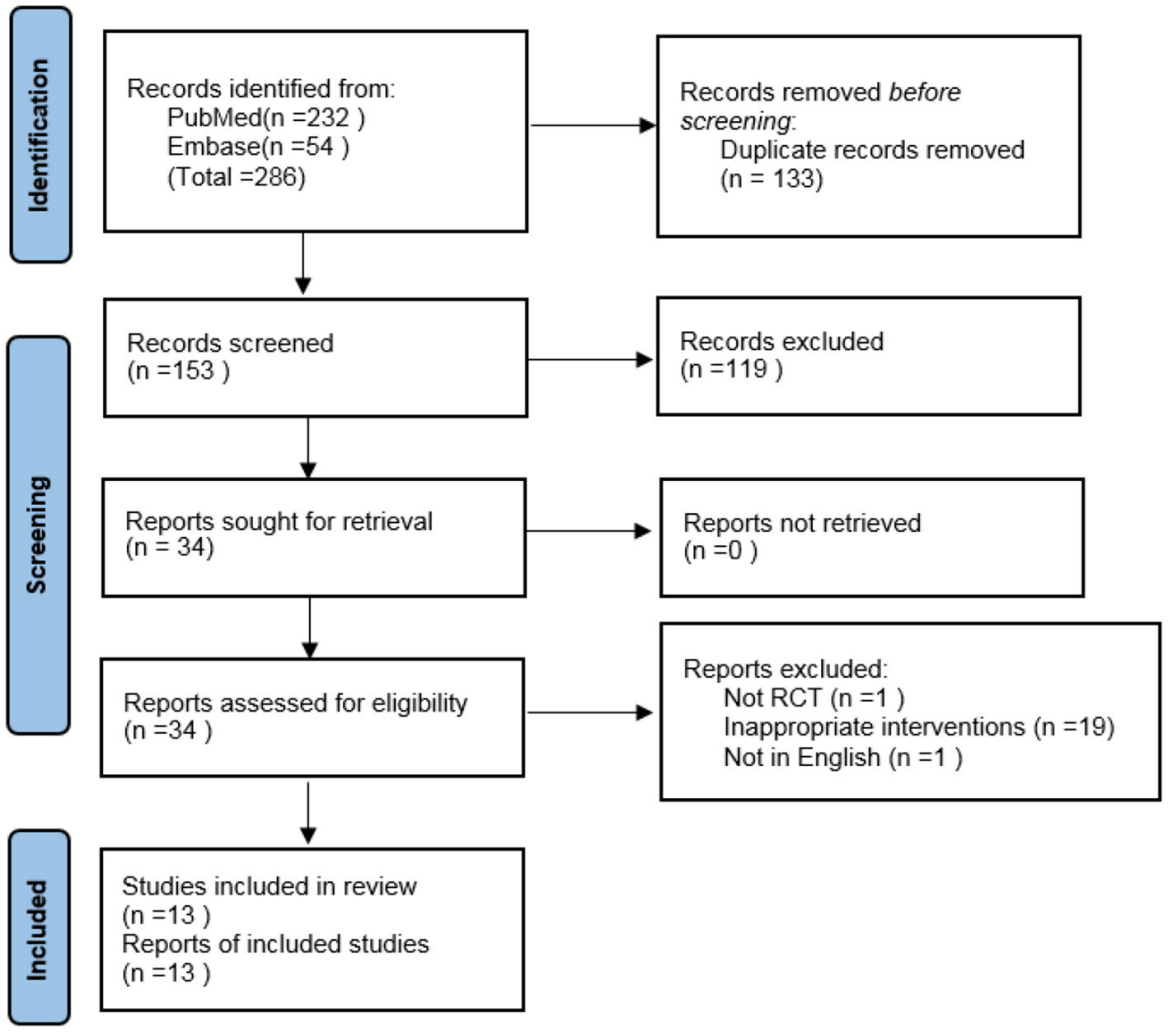
Flowchart of the literature search and selection process.

Using the specified search strategy, 286 references were initially retrieved. After removing duplicate articles, 153 articles remained. Of these, 13 met both the inclusion and exclusion criteria, and were included in the meta-analysis (9, 12–23). The screening process is shown in Figure 1.

### 3.2. Characteristics of included studies

All 13 RCTs were conducted outside of China and were published in English. The basic characteristics of the RCTs are listed in Table 1.

**Table 1:** Basic characteristics of the included studies.

**Study**	**Country**	**Publication language**	**Number of cases (N/P)**	**Average age (N/P, years)**	**Intervention measures**		**Follow-up time**	**Outcome**
					**N (pieces)**	**P (liters)**		
Aronchick (9)	U.S.A.	English	99/100	57.6/58.8	24 or 32	4 L	From taking laxative to end of colonoscopy	①
Kastenberg (12)	U.S.A.	English	420/425	55.8/57	40	4 L	From taking laxative to end of colonoscopy	①
Kastenberg et al. (15)	U.S.A.	English	420/425	56/57	40	4 L	From taking laxative to end of colonoscopy	②③④
Lichtenstein et al. (16)	U.S.A.	English	205/206	55.9/56.3	32	2 L	From taking laxative to end of colonoscopy	④
Johanson (13)	U.S.A.	English	205/206	55.9/56.3	32	2 L	From taking laxative to 2–4 days after colonoscopy	①④
Aihara (14)	Japan	English	10/10	49.42/51.58	Maximum 50 grams	Maximum4 L	From taking laxative to 7 days after colonoscopy	⑤
Hosoe et al. (17)	Japan	English	41/45	56.5/61.0	50 grams	2 L	From taking laxative to end of colonoscopy	②③
Kambe et al. (18)	Japan	English	44/48	58.0/56.6	50 grams	2 L	From taking laxative to end of colonoscopy	①
Jung et al. (19)	Korea	English	158/162	46.5/48.6	32	4 L	From taking laxative to end of colonoscopy	①③
Seung-Hwa (20)	Korea	English	32/30	40.4/40.6	32	4 L	From taking laxative to end of colonoscopy	②④
Kumagai (21)	Japan	English	48/45	45.2/46.2	30	Maximum 2 L	From taking laxative to end of colonoscopy	①④
Ako et al. (22)	Japan	English	95/98	50/49.5	30	2 L	From taking laxative to end of colonoscopy	①③
Chaussade et al. (23)	France	English	226/226	54/51.5	32	4 L	From taking laxative to end of colonoscopy	①②

N, sodium phosphate tablets; P, polyethylene glycol lavage solution; ① Excellent and good intestinal cleaning quality rating; ② Incidence of nausea; ③ Excellent and good taste rating; ④ Willingness to repeat treatment rating; ⑤ Changes in serum electrolytes after intestinal preparation.

### 3.3. Quality evaluation of included studies

**Figure 2:**
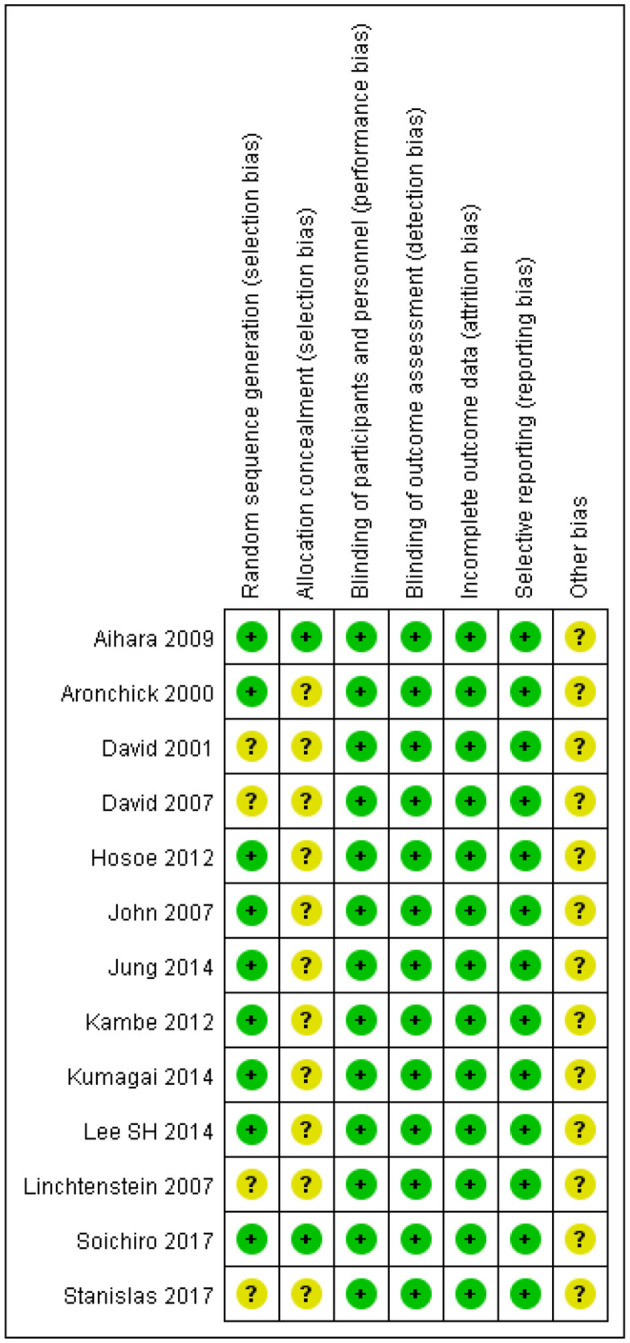
Quality evaluation of included studies.

The quality of the 13 RCTs is shown in Figure 2. All articles mentioned using a “random” design but only ten described the specific randomization method used. Only two of 13 trials described the allocation plan. All trials used a blinded method to evaluate research objects and outcomes, had complete outcome data and did not describe other potential biases.

### 3.4. Results of meta-analysis

#### 3.4.1. Adequate cleansing quality

**Figure 3:**
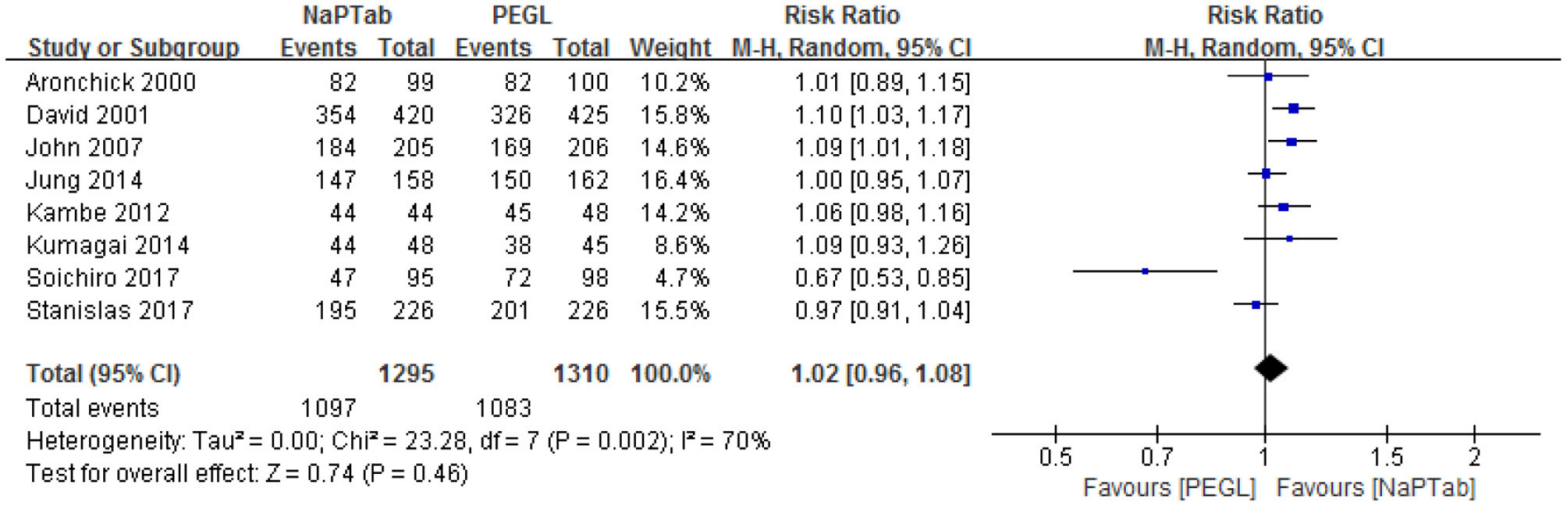
Rate of excellent and good bowel cleaning quality.

Eight trials including 2,605 patients used adequate cleansing quality as an index of bowel cleaning quality (the rate of excellent and good) (9, 12, 13, 18, 19, 21–23). Heterogeneity testing revealed significant heterogeneity among the RCTs (*P* = 0.002), so the random effect model was used for analysis. Meta-analysis results showed that the ratio of excellent and good bowel cleaning quality was similar between the NaPTab and PEGL groups [RR 1.02, 95% CI (0.96–1.08), *P* = 0.46] (Figure 3).

**Figure 4:**
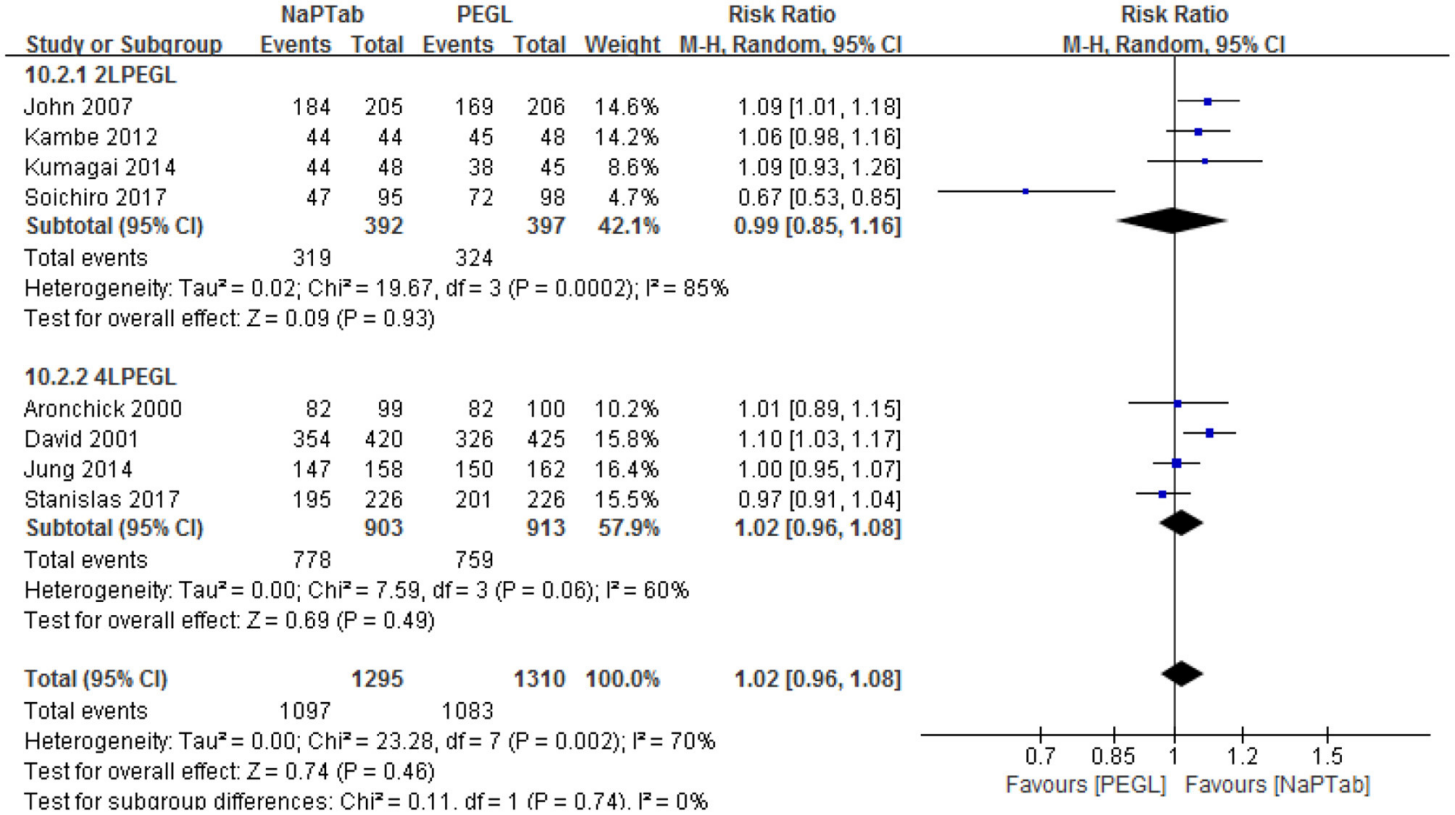
Subgroup analysis according different volumes of PEGL.

Subgroup analysis according to different volumes of PEGL was conducted, 2 L PEGL subgroup and 4 L PEGL subgroup. Heterogeneity testing revealed significant heterogeneity among each subgroup, so the random effect model was used for analysis. Meta-analysis results showed that in each subgroup the ratio of excellent and good bowel cleaning quality was similar between the NaPTab and PEGL groups [*P* = 0.93, *P* = 0.49 accordingly] (Figure 4).

**Figure 5:**
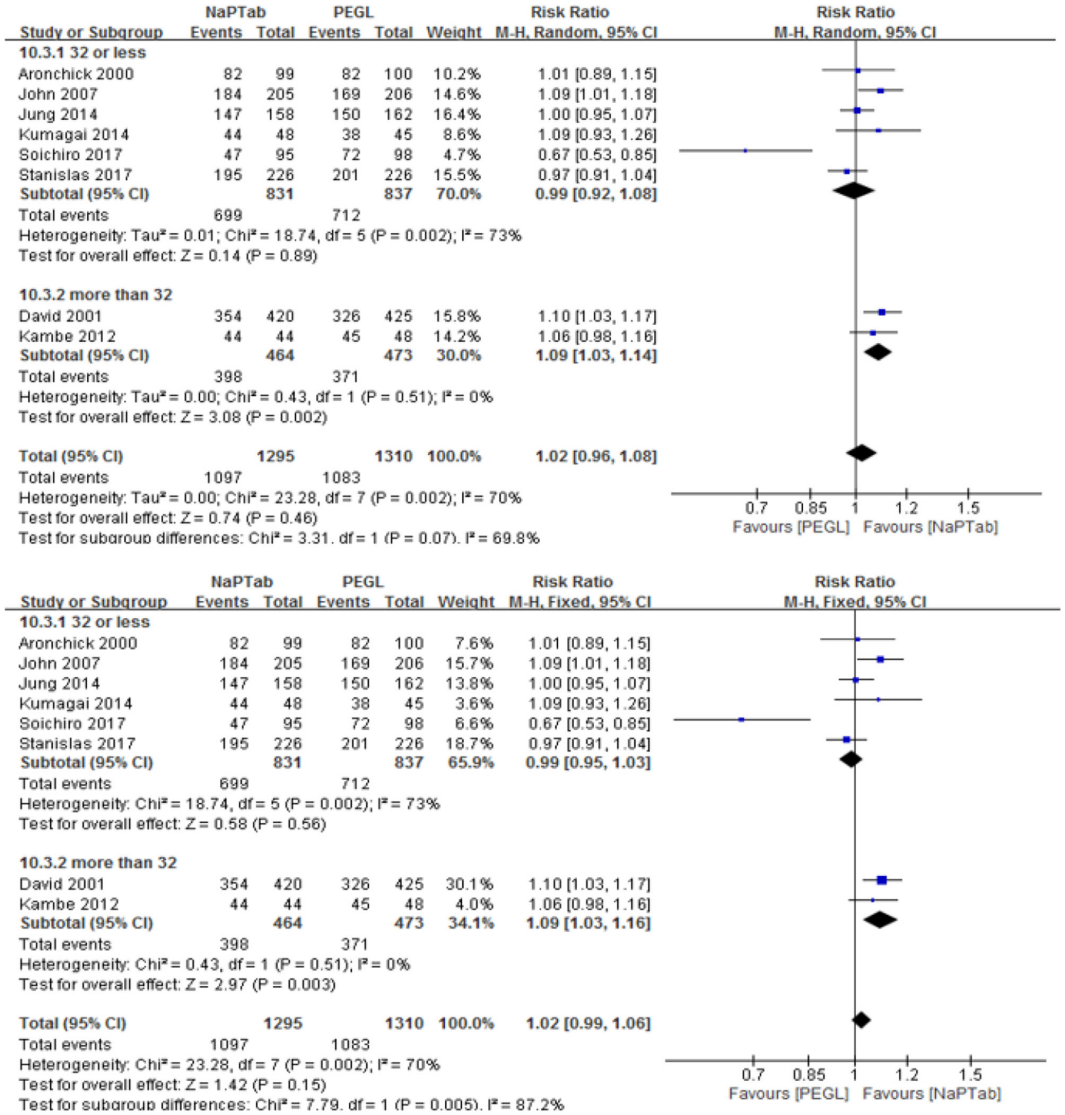
Rate of excellent and good bowel cleaning quality.

Subgroup analysis according to different numbers of sodium phosphate tablets was conducted, 32 or less subgroup and more than 32 subgroup. Heterogeneity testing revealed significant heterogeneity among 32 or less subgroup, so the random effect model was used for analysis. Meta-analysis results showed that in 32 or less subgroup the ratio of excellent and good bowel cleaning quality was similar between the NaPTab and PEGL groups [*P* = 0.89]. However, heterogeneity testing revealed no significant heterogeneity among more than 32 subgroup, so the fixed effect model was used for analysis. Meta-analysis results showed that in more than 32 subgroup the ratio of excellent and good bowel cleaning quality of the NaPTab group was higher than the PEGL group [RR 1.09, 95% CI (1.03, 1.16), *p* = 0.003] (Figure 5).

#### 3.4.2. Nausea

**Figure 6:**
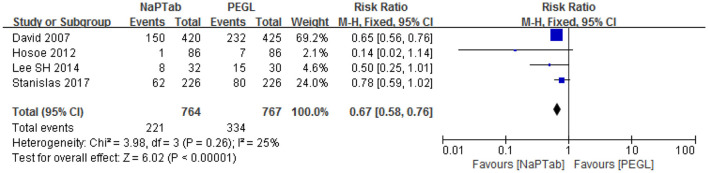
Incidence of nausea.

Four trials including 1,531 patients used nausea incidence as an indicator of side effects (15, 17, 20, 23). Heterogeneity testing showed no significant heterogeneity among the RCTs (*P* = 0.26) so the fixed effect model was used for analysis. Meta-analysis results showed that nausea incidence was lower in the NaPTab group than in the PEGL group [RR 0.67, 95% CI (0.58–0.76), *p* < 0.00001] (Figure 6).

#### 3.4.3. Taste evaluation

**Figure 7:**
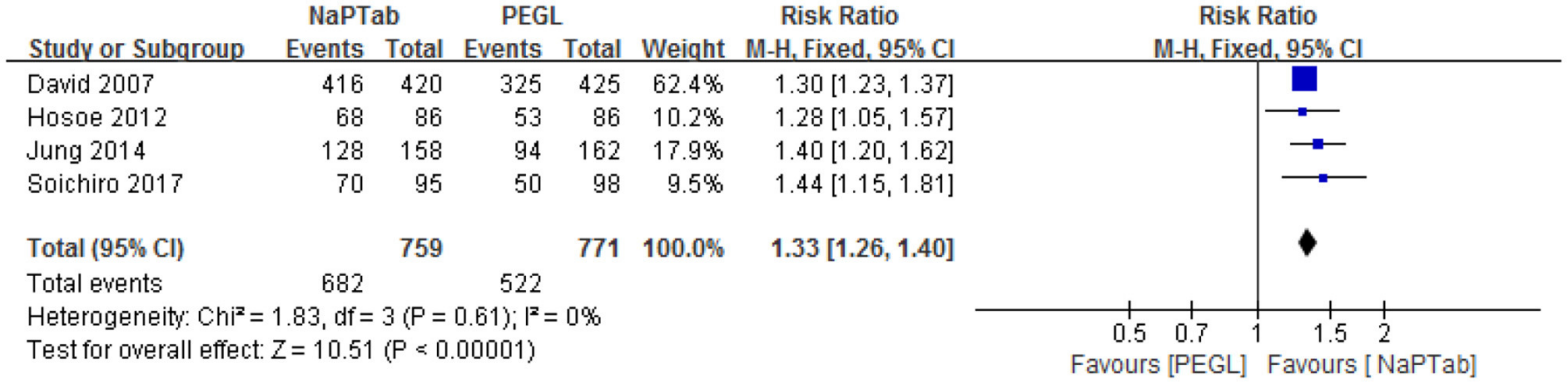
Rate of excellent and good taste.

Four trials including 1,530 patients used the rate of excellent and good taste to compare the two preparations (15, 17, 19, 22). Heterogeneity testing showed no significant heterogeneity among the RCTs (*P* = 0.61), so the fixed effect model was employed for analysis. Meta-analysis results showed that the taste rating of the NaPTab group was higher than the PEGL group [RR 1.33, 95% CI (1.26, 1.40), *p* < 0.00001] (Figure 7).

#### 3.4.4. Willingness to repeat treatment

**Figure 8:**
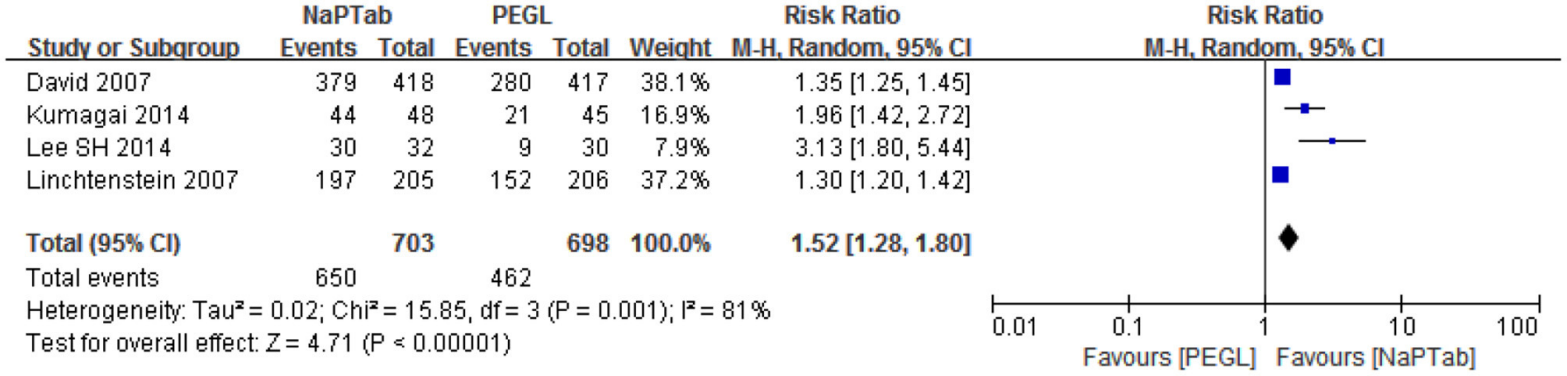
Willingness to repeat treatment.

Four articles including 1,401 patients assessed willingness to receive the same bowel preparation method (15, 16, 20, 21). Heterogeneity testing showed that there was significant heterogeneity among the RCTs (*P* = 0.001), so the random effect model was used for analysis. Meta-analysis results showed that the NaPTab group had a higher willingness to repeat the treatment than the PEGL group [RR 1.52, 95% CI (1.28–1.80), *P* < 0.00001] (Figure 8).

#### 3.4.5. Serum electrolyte measurements

##### 3.4.5.1. Serum potassium

**Figure 9:**
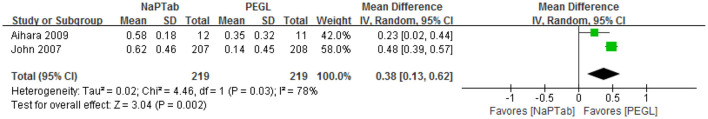
Changes in serum potassium (mEq/L).

Two articles including a total of 438 patients assessed the change in serum potassium levels before and after colonoscopy preparation (13, 14) and found that levels decreased in both groups. Heterogeneity testing showed that there was significant heterogeneity among the RCTs (*P* = 0.03) so the random effect model was used. Meta-analysis results showed that the NaPTab group had a greater decrease in serum potassium than the PEGL group [MD = 0.38, 95% CI (0.13–0.62), *P* = 0.006] (Figure 9).

##### 3.4.5.2. Blood calcium

**Figure 10:**
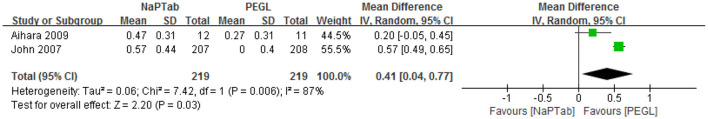
Changes in blood calcium (mg/dL).

Two articles including a total of 438 patients assessed the change in serum calcium before and after colonoscopy preparation (13, 14) and found that levels decreased in both groups. Heterogeneity testing revealed significant heterogeneity among the RCTs (*P* = 0.006) so a random effect model was used. Meta-analysis results showed that the decrease in serum calcium was greater in the NaPTab group than in the PEGL group [MD = 0.41, 95% CI (0.04–0.77), *P* = 0.03] (Figure 10).

##### 3.4.5.3. Blood phosphorus

**Figure 11:**
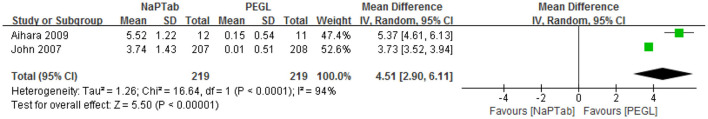
Changes in blood phosphorus (mg/dL).

Two articles including 438 patients assessed the change in serum phosphorus (inorganic) before and after colonoscopy preparation (13, 14). While patients in the NaPTab group had high serum phosphorus levels after preparation, some patients in the PEGL group had increased serum phosphorus levels while others had decreased levels. Heterogeneity testing revealed significant heterogeneity among the RCTs (*P* < 0.0001) so the random effect model was employed. Meta-analysis results showed that the NaPTab group had a greater increase in serum phosphate than the PEGL group [MD 4.51, (95% CI 2.9–6.11), *P* < 0.00001] (Figure 11).

### 3.5. Sensitivity analysis and publication bias

**Figure 12:**
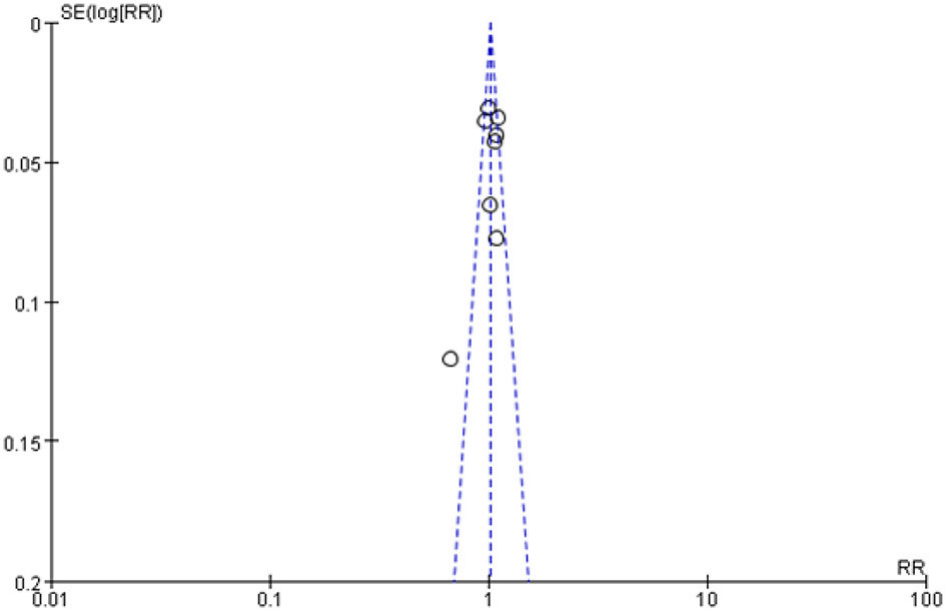
Funnel chart of bowel cleaning quality.

A sensitivity analysis of adequate cleansing quality by omitting one study at a time did not fundamentally influence the pooled RR, suggesting that the combined RR was valid and credible. No evidence of publication bias was observed when the difference of adequate cleansing quality between NaPTab and PEGL were assessed (Figure 12).

## 4. Discussion

Colonoscopy has been used in medicine for over half a century. Many primary hospitals are now implementing this technique since it allows direct examination of the colonic mucosa. However, inadequate bowel preparation can make it difficult to clearly observe mucous membranes (3), resulting in missed diagnosis, or wasted time repeating the examination. Inadequate intestinal preparation can also increase the risk of intestinal perforation and peritonitis. If the intestine still includes a high amount of feces, high-frequency electronic resection can risk igniting any intestinal gas and result in explosion (24). A systematic review of 18 randomized controlled trials comparing sodium phosphate and polyethylene glycol solution was published in 2005. This review showed no differences in intestinal cleaning quality between the two groups. Sub-group analysis showed that the intestinal cleaning quality of NaPTab was significantly higher than PEGL (25). However, only 1,044 cases were included and no quantitative analysis of adverse reactions, patient acceptance, or electrolyte changes were assessed.

The current study collected 13 RCT studies that compared the success of intestinal preparation using NaPTab or PEGL prior to colonoscopy. Eight trials including 2,605 patients compared the effects of intestinal cleaning. Meta-analysis results showed that the NaPTab group was more likely to have an excellent and good rating of intestinal cleaning quality than the PEGL group (RR 1.02, 95% CI 0.96–1.08); however, the difference was not significant. Subgroup analysis showed that the NaPTab subgroup of more than 32 had a higher excellent and good rating of intestinal cleaning quality than the PEGL group (*p* = 0.003), but only two studies was included. The NaPTab group also had a lower incidence of nausea, a higher excellent and good taste rating and a higher willingness to repeat the treatment than the PEGL group, and these differences were significant. Two trials including 438 patients detected changes in serum potassium, calcium, and phosphorus levels before and after intestinal preparation. Serum potassium and calcium levels decreased after intestinal preparation with both treatments but the change was statistically greater for the NaPTab group than the PEGL group. Meanwhile, blood phosphorus increased following both treatments but the rise was significantly higher for the NaPTab group.

Currently, there are both oral sodium phosphate solutions and tablets available on the market. This treatment provides a low-capacity method of intestinal preparation and is convenient for patients to use. NaPTab also has a good taste, is well-tolerated and has a similar effect on intestinal cleaning than PEGL. Subgroup analysis showed that higher dose NaPTab may have better cleaning effect. Constipation is an important and highly common risk factor for poor quality of intestinal preparation (26). At present, there is little consensus on bowel preparation for constipation patients. A recent meta-analysis found that OSP may result in superior colonic cleanliness when compared to PEGL, however, quality of evidence was low (27). OSP may be a better choice for patients with chronic constipation and colonic diverticulum. The reason may be that OSP is a hyperosmotic agent, which can cause intestinal dilatation, peristalsis and evacuation of intestinal contents by inhaling water into the lumen of bowel (28).

Polyethylene glycol based intestinal lavage is an isosmotic solution that passes through the bowel without absorption or secretion. PEGL has been safely used in patients with serum electrolyte imbalance, advanced liver insufficiency, acute and chronic renal failure and congestive heart failure. PEGL does not change the histological characteristics of colon mucosa and can be used in patients suspected of having inflammatory bowel disease without obscuring the diagnostic capabilities of colonoscopy or biopsy analysis. However, patients have to take large volumes of fluid in order to achieve a cathartic effect, and the taste of PEGL is poor. In order to avoid the problem of volume and taste, a PEGL containing ascorbic acid (ACS) was developed. Large doses of ascorbic acid that are not fully absorbed remain in the colon cavity, where it plays an osmotic role, so a small amount of PEG is required (29).

Changes in electrolyte levels are more obvious for patients in the NaPTab group after intestinal preparation. This is a particular concern for high blood phosphorus, which is associated with severe kidney damage. A study identified 21 patients with acute renal failure a few weeks after a colonoscopy preparation using OSP. While renal function improved for most patients, four patients required renal replacement therapy (30). In addition, between January 2006 and December 2007, the Food and Drug Administration (FDA) reported 171 patients with acute renal failure after using OSP. The Huashan Hospital of Fudan University described a 61-year-old male who suffered from acute phosphate nephropathy (APN). His serum creatinine was normal before the colonoscopy but increased to 248 μmol/L within 4 months after taking OSP. Von Kassa staining of his renal pathology indicated APN (31). Another study found that the risk of acute renal failure was three times higher for OSP than PEGL. After nearly 9 months, the serum creatinine levels of most patients did not return to baseline levels (32). The US FDA has published high risk factors associated with acute renal failure from sodium phosphate including advanced age, hypovolemia, acute and chronic kidney disease, using diuretics and other drugs that influence renal blood flow, and ACEI/ARB. Advanced age and renal function were also highly correlated with APN (33). High blood pressure (30) and inflammatory bowel disease (IBD) (34) are predisposing factors. Drugs that alkalize urine can decrease the precipitation threshold of calcium phosphate, so are was not recommended for use with OSP (35). OSP for bowel preparation was not recommended by the 2019 Guide of the European Society of Gastroenterology (ESGE) for routine use, but the quality of support studies was poor (36). While patients do not favor PEGL for intestinal preparation because of its poor taste and large oral capacity, this treatment carries a lower risk of electrolyte disorders and kidney damage than sodium phosphate and is preferred over OSP for high-risk patients (37).

There are various ways for bowel preparation before colonoscopy, and different population need different volume of lavage or dose possibly. Westerners are heavier in body weight and need larger in dose when preparing. In addition, many cases require individualized bowel preparation based on risk factors. There are many risk factors for poor intestinal preparation, such as history of abdominal surgery. There are some possible explanations for the poor intestinal preparation in patients with a history of abdominal surgery. First of all, the patient has a decreased intestinal motility after intestinal resection. Second, after abdominal surgery, intra-abdominal adhesion may lead to the fixation of flexible intestine, which may lead to local retention of fecal substances. A study shown that gastric/small intestinal surgery was a potential risk factor for poor bowel preparation (38). According to the different conditions of abdominal surgery, except for using appropriate amount of intestinal cleanser, we can also choose prokinetic drugs, diet restrictions and other measures. Increased BMI is not predictive of poor bowel preparation for colonoscopy (39). In fact, the opposite is true for a particular group of patients, as it shows that the quality of bowel preparation is reduced for underweight females who are older than 70 years and have constipation, thus causing unfavorable colonoscopy outcomes (40). For such patients, if she has no risk factors of electrolyte disorder and acute phosphate nephropathy, she can consider using OSP. If yes, consider PEGL-ACS.

In conclusion, the current meta-analysis showed that NaPTab and PEGL have a similar cleaning effect before colonoscopy and that the tolerance of NaPTab is superior to PEGL. However, NaP tablets have a stronger effect on serum potassium, calcium and phosphorus levels. For patients with low potassium, low calcium and renal insufficiency, NaP tablets should be prescribed with caution. For patients at high risk for acute phosphate nephropathy, NaP tablets should be avoided. These conclusions are limited by both the number and quality of included studies and require verification by larger and more high-quality studies. Individualized bowel preparation based on risk factors is required for special cases. Good cleaning effect, low capacity, good taste, few adverse reactions and high security are goals to consider for the design of future intestinal preparations before colonoscopy. More clinical trials are required to evaluate relevant drugs or methods.

## Data availability statement

The raw data supporting the conclusions of this article will be made available by the authors, without undue reservation.

## Author contributions

All authors listed have made a substantial, direct, and intellectual contribution to the work and approved it for publication.
